# Megatrends and Trends Shaping Supply Chain Innovation

**DOI:** 10.1007/978-3-030-63505-3_1

**Published:** 2020-10-23

**Authors:** Dimitra Kalaitzi, Aristides Matopoulos, Rosanna Fornasiero, Saskia Sardesai, Ana Cristina Barros, Sébastien Balech, Victoria Muerza

**Affiliations:** 2 STIIMA-CNR, Milano, Italy; 3 Fraunhofer IML, Dortmund, Nordrhein-Westfalen Germany; 4 INESC TEC, Porto, Portugal; 5 Aston University, Birmingham, UK; 6 Department of Engineering Systems & Supply Chain Management, College of Engineering and Physical Sciences, Aston University, Aston Triangle, Birmingham, B4 7ET UK; 7 Institute of Intelligent Industrial Technologies and Systems for Advanced Manufacturing, National Council of Research (STIIMA-CNR), Via Alfonso Corti, 12-20133 Milan, Italy; 8 Institute of Electronics, Computer and Telecommunication Engineering, National Council of Research (IEIIT-CNR), c/o Università di Padova, via Gradenigo 6/B, 35131 Padova, Italy; 9 Fraunhofer Institute for Material Flow and Logistics, Joseph-von-Fraunhofer-Str. 2-4, 44137 Dortmund, Germany; 10 INESCT TEC Institute for Systems and Computer Engineering, Technology and Science, Campus da FEUP, Rua Dr. Roberto Frias, 4200-465 Porto, Portugal; 11 PNO CONSULTANT, Avenue de la Joyeuse Entrée 1, 1040 Brussels, Belgium; 12 MIT International Logistics Program, Zaragoza Logistics Center, C/Bari 55, Edificio Náyade 5 (PLAZA), 50197 Saragossa, Spain; 13 University of Zaragoza, Quantitative Methods for Business and Economy, Gran Vía 2, 50005 Saragossa, Spain

**Keywords:** Megatrends, Trends, PESTLE analysis, Logistics, Supply chain management, Supply chain scenarios

## Abstract

Companies operate in a macro-environment that is changing considerably due to large, transformative global forces namely megatrends and trends. The wave of these megatrends and trends generates new prospects as well as challenges for the future of supply chains. This chapter provides a review of 23 major megatrends and 72 trends identified in multiple dimensions along Political, Economic, Social, Technological, Legal, and Environmental (PESTLE) dimensions. The results are based on a systematic literature review and an experts’ workshop, and can be used to generate future supply chain scenarios.

## Introduction

Megatrends are “large social, economic, political and technological changes [that] are slow to form, and once in place, they influence us for some time” (Kotler 2002, p. 137). Trends, as an indication into a direction of change, are driven by megatrends. For example, aging population is a megatrend and some of its trends are buying habits, and workforce gaps. Megatrends are observed over decades (15 + years) and affect almost all world regions while trends are observed over 5–10 years on specific parts of the world (Pictet asset management 2017).

Megatrends (e.g. new digital technologies) have a heavy influence on supply chains and they could impact product, process and network configuration (e.g. Calatayud et al. 2019; Birkel and Hartmann 2019). For example, blockchain technology (which is a trend) could lead to transparency, but it could disrupt the structure of supply chains as some intermediaries may have to be removed and the supply chain finance (Wang et al. 2019). Supply chain finance solutions can also be reshaped and boosted by the use of other technological trends e.g. artificial intelligence, internet of things, and big data analytics (Caniato et al. 2019).

Given the presence of these technologies, the role and relevance of supply chain governance mechanisms will be affected; e.g. digital transformation impacts on supply chain collaboration and integration (Barbieri et al. 2019). Companies have to develop a thorough understanding of these changes to avoid unexpected and negative effects and concurrently create opportunities to ensure long term survival. Future scenarios and hypotheses can be developed that will explore about how specific industries might evolve (Gernandt 2012).

There are numerous studies (e.g. Gernandt 2012; Seppälä 2016) that have identified several megatrends and trends such as electric vehicles, efficient multi-modal networks (e.g. Speranza et al. 2018), knowledge based development to talent management (e.g. Bowersox et al. 2000; Stank et al. 2015; Sweeney 2013), additive manufacturing and information systems (Fawcett and Waller 2014; Kemppainen and Vepsäläinen 2003). There is a lack of state of the art and comprehensive review of the list of megatrends and associated trends in the field of supply chain management and logistics. Apart from the studies mentioned above, consulting firms such as EY, PWC and KPMG have explored megatrends by frequently mentioning demographic shifts, geopolitical changes and environmental impacts as key megatrends. These studies lack comprehensive and coherent understanding of megatrends with political, economic or social megatrends being the center of attention (Malik and Janowska 2018).

Thus, the motivation of this study is to identify and verify systematically which megatrends are considered the most influential in this field by researchers and professionals. The purpose of this chapter is to recognize and increase knowledge of the megatrends shaping the future of supply chains and provide a comprehensive list of trends that addresses critical dimensions e.g. political, technological, environmental, and others. Through a systematic literature review, the following research questions intend to be answered in this chapter:

*RQ1: “What are the megatrends shaping the future of the supply chains?”*

*RQ2: “What are the trends that can be derived from these megatrends shaping the future of supply chains?”*

From a managerial perspective, this analysis allows companies to build a long-term supply chain strategy that is proactive, rather than reactive, by having a thorough understanding of their current position, but also ensuring they have a plan to respond in complex and dynamic environments and reduce perceived uncertainty. This research further provides theoretical contribution by filling the literature review gap as a comprehensive list of megatrends and trends that will be identified. Researchers can focus on each of the megatrends and its implications and develop supply chain scenarios. Thus, the results of this chapter will be further used in the next chapters as a basis for future scenarios generation. The remainder of this chapter is organized as follows: Sect. 2 outlines the research design; Sect. 3 presents and discusses the findings of the literature review. Section 4 draws conclusions and discusses future research avenues.

## Methodology

A systematic literature review and a workshop with experts serves to identify relevant megatrends and trends for supply chain management. According to Denyer and Tranfield (2009), the systematic literature review process entails five steps: (1) process question formulation, (2) locating literature, (3) study selection and evaluation, (4) analysis and synthesis, and finally (5) reporting and using the results. The steps are described below, apart from the first step as it has been already discussed in the previous sub-section and the final step, i.e. reporting and using of the results, which is presented in the next sub-section.

The search of the studies was conducted from November 2017 to January 2018. The following keywords were used to find the relevant studies: (supply chain management) OR (logistics) OR (manufacturing) OR (transportation) OR (process manufacturing) OR (distribution) AND (future supply chain) OR (next generation supply chains) OR (megatrends) OR (trends).

Relevant journal papers and book chapters were identified by using the search field “Topic” to screen well-known academic databases namely ABI/INFORM Global, Academic Search Premier (EBSCO), Science Direct, Emerald and Google Scholar. Our research was not limited to specific areas e.g. “Engineering”, “Operations Research Management Science”.

Apart from journal papers and book chapters, grey literature that is not controlled by commercial publishers and entails government, academics, business and industry in print and electronic formats supplements the literature review. The grey literature can minimize bias, “increase reviews’ comprehensiveness and timeliness, and foster a balanced picture of available evidence” (Paez 2017, p. 233). Grey literature identified by using web searching, particularly the Google search engine was utilized (file type: pdf search filter, as most of the reports, conference papers etc. are in the form of PDFs) or search within certain websites was also followed e.g. PricewaterhouseCoopers, Roland Berger websites to identify information regarding a specific megatrend or trend e.g. resource scarcity, climate change.

The risk of bias was reduced by implementing clear exclusion and inclusion criteria. As our search shall support developments of the future, we only integrated studies from 2010 onwards. The following filters were used to include the most relevant studies: title, abstract and keywords, introduction, conclusion were checked. Duplicate studies were excluded as well as articles or reports that are irrelevant and not written in English, leading to the review of 125 studies. Multiple databases were used, thus the authors contributed with different relevant articles to minimize bias (Kalaitzi et al. 2018a).

**Fig. 1:**
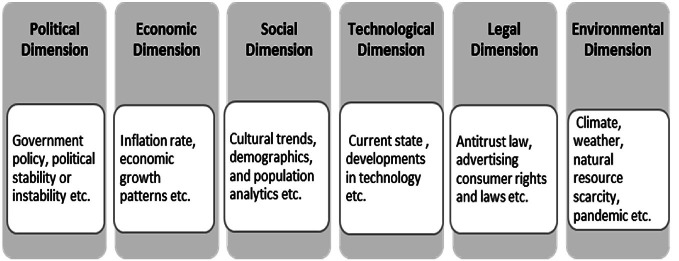
PESTLE dimensions. Adapted from Yudha and Tjahjono (2019)

The data was synthesized via thematic analysis using the iterative thematic synthesis process and the NVivo software. ‘Open coding’ was used to identify trends within the 349 studies and thus create the first-order themes, the trends. According to Collis and Hussey (2009, p. 179) *“The codes are labels which enable the qualitative data to be separated, compiled and organised”*. Then the second-order themes were created, which are the megatrends, by connecting the first-order themes. The last step was to link all the related second-order themes under one aggregate dimension, namely Political, Economic, Social, Technological, Legal and Environmental (PESTLE). By utilizing the PESTLE analysis all the external forces/factors in the macro environment can be captured. Managers in the field of supply chain can use these factors to identify where supply chains currently stand and assess their implications on the future development of their business. A similar approach has been applied in other studies e.g. Yudha and Tjahjono (2019) who analyzed renewable energy industries to identify inefficient legislations and support the development of new strategic policies in the fossil fuel industry. Figure 1 describes the PESTLE dimensions and its content as used by Yudha and Tjahjono (2019).

For example, in the political dimension two second-order themes emerged, namely protectionism (first-order themes: import tariffs, quotas, different tax structures) and political stability (first-order themes: terrorism/conflicts). To identify the first-order themes, the following procedure was applied: the authors initiated a data extraction via randomly selecting a sample of studies to keep the data extraction consistency. The final findings were checked for consistency at a meeting that was arranged to discuss and resolve any disagreements.

**Fig. 2:**
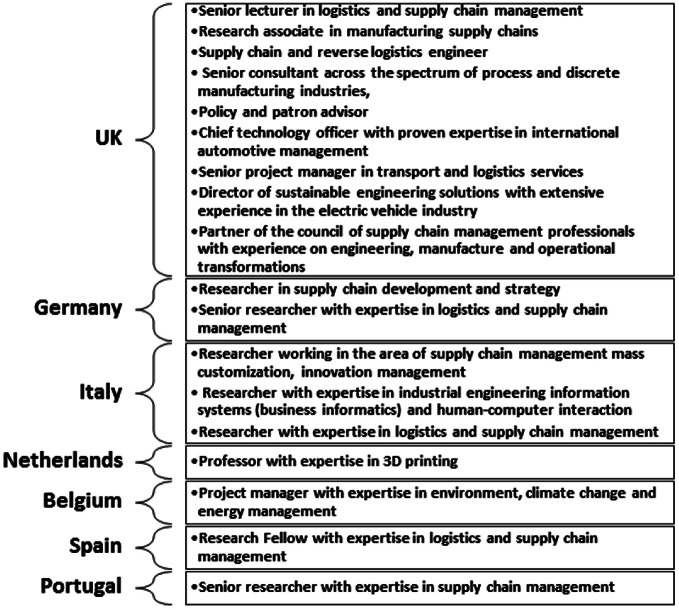
Workshop participants across seven countries

After the systematic literature review, a workshop with 18 academics and practitioners from the UK, Germany, Italy, Netherlands, Belgium, Spain and Portugal was held (see Fig. 2). A mind mapping software supported the discussion to refine and extend the findings of the megatrends and trends gained from the literature review. An expert’s workshop is a cost-effective and time efficient method in comparison with others such as Delphi research; as the experts gather at the same time and location, it maximizes the compilation of information. It thus leads to richness of information, and enable the experts to participate actively. The workshop participants for the conducted expert workshop are listed in Fig. 2.

## PESTLE Analysis

Based on the thematic analysis of the secondary and primary data, 23 megatrends and 72 trends emerged which are further explored and presented in the below sub-sections based on the aggregate PESTLE dimensions.

### Political

One of the most important political changes emerging over the last years is protectionism including several trade policies such as tariffs and import quotas, thus policies that try to protect domestic industries against foreign competition. For example, European Union (EU) duty on imports cars from the United States (US) is 10%, whereas the US duty on imports from the EU is only 2.5% (Europa 2015). Another example of protectionism is the US import tariffs of 25% on steel and 10% on aluminum. Companies operating in Mexico and China may be affected by increasing import duties and tariffs (PwC 2017). Regarding quotas, China applied those for rare earth elements from 2005 until 2015 until the World Trade Organization forced China to remove the export quotas. Apart from tariffs and quotas, governments offer subsidies (e.g. cheap loans) to local firms so they can compete well against foreign imports (de Kluyver 2010). For example, the EU offers more than €112 billion per year of fossil fuel subsidies to the transportation industry (Hill 2017). Last but not least, it needs to be highlighted that the total tax share in consumers’ prices and taxation systems are not the same across the globe and thus integrate complexity into the supply chain. For instance, different measures to calculate tax are used in Bangladesh, where taxation for tyres is based on retail prices whereas cigarette length is taken into account in India (Shang et al. 2015).

Another megatrend is related to political stability, an indicator of growth, which is crucial for companies. In contrast, political instability (i.e. terrorism/conflicts such as wars, social unrest) can have severe impacts on supply chain operations. For instance, the attack in the twin towers of New York City in September 11, 2001 led Ford, an automotive manufacturer, to shut down five of its plants in the USA and to deficit in land imports exceeding US $350mln (Bueno-Solano and Cedillo-Campos 2014). Regarding social unrest, all regions face some degree of instability, e.g. as anti-government demonstrations, riots, and strikes. For example, “labor issues at West Coast container ports disrupted service in 2015, drove volumes to other ports, and altered seasonal patterns” (Kearney ΑΤ 2016a, p. 18). Finally, the megatrend supranationalism is linked with trade agreements and free movement which determine supply chain decisions (e.g. the number and location of the production facilities, supplier selection). Brexit provides an example for this megatrend with a direct impact on supply chains. Specifically, as trade agreements will be restricted some studies highlight that 32% of EU suppliers will be substituted by local suppliers and 46% of EU companies will avoid UK suppliers (McKevitt 2017). Table 1, provides an overview of the megatrends and trends related to the political dimension.

**Table 1:** Political dimension megatrends and trends

Megatrends	Trends	References
Protectionism	Import tariffs, quotas, subsidies different tax structures	Benjelloun et al. (2009), Boston Consulting Group (2016), BVL (2013), Clausen et al. (2014), Deloitte (2015a), DHL (2013, 2016a, 2017) Hajkowicz et al. (2016), Deloitte (2015b), Harrington (2015), Kewill (2013), PwC (2009), Schuckmann et al. (2012), SMMT (2015), von der Gracht and Darkow (2011)
Political stability	Terrorism/conflict, social unrest	BVL (2013), Cerasis (2017), DHL (2012), FTA (2016, 2017), Grant Thornton (2017), PwC (2011, 2017), Sana (2017), von der Gracht and Darkow (2011), World Bank Group (2017)
Supranationalism	Trade agreements, free movement	KPMG (2014, 2017), WEFORUM (2016), World Economic Forum (2016)

### Economic

A key trend that emerged under the economic dimension is economic growth in countries such as China and the MINT (Mexico, Indonesia, Nigeria, and Turkey) results in global trade shift (Francesco and Ardita 2015). It is projected that by 2030, China will be the largest economy in terms of Gross Domestic Product (GDP) while by 2050 India would be the third largest economy in the world. This will influence the demand, and the design of the supply chain networks (PwC 2015). Glocalization is another trend that entails the process of adapting international products to the local culture in which they are sold and also impact supply chain configuration and product structure (Hong and Song 2010). For example, fast-food chains such as McDonald offer their global, iconic brands but at the same time there are local menu options.

Moreover, digital economy trend appeared through the literature where customers use online platforms (e.g. Airbnb, Uber) to facilitate the process of sharing. Sharing economy industry meanwhile established a worth of £7 billion a year (Kleiner Perkins 2017). Peer-to-peer transportation facilitated transactions of €5 billion in 2015 (PwC 2016a) and it can be applied to inter/ intra- city logistics and movement of goods by ride-sharing or by truck-sharing (DHL 2016a). Regarding the trend from an economy of goods to an economy of services, new selling platform or new, upcoming internet services will be introduced. For example, grocery stores with both virtual and physical presence are introduced by platform providers e.g. by Amazon and Alibaba. Concerning the megatrend financial innovation, there are trends such as cashless payments and digital currencies that aim to improve the security of the financial transactions in the supply chains. Thus, business-to-consumer (B2C) transactions are increasingly digitized (Bons et al. 2012). Last but not least, financial technology (Fintech) firms are expected to revolutionize supply-chain finance. However, FinTech companies face regulatory uncertainty that can act as burden on companies’ prospects for growth (Boston Consulting Group 2017).

Thus, under the economic dimension three megatrends and 11 trends emerged (see Table 2).

**Table 2:** Economic dimension megatrends and trends

Megatrends	Trends	References
Global trade shift	Economic growth in emerging economies, export growth, investment, globalization, glocalization and emergence of born-global firms	Accenture (2016), Aggelakakis et al. (2015), ALICE (2013), BVL (2013), Kearney ΑΤ (2014, 2016a), Boumphrey and Brehmer (2017), Cerasis (2017), Clausen et al. (2014), Csiro (2014, 2016), Deloitte (2017), DHL (2015, 2016a, 2017), Elmegaard (2016), European Agency for Safety and Health at Work (2016), FTA (2016), Gernandt (2012), Hajkowicz et al. (2016), Harrington (2015), ITC (2017), McKinsey Global Institute (2012, 2016a, 2016b), PwC (2015, 2016b, 2011), Roland Berger (2014), Sana (2017), Schuckmann et al. (2012), SMMT (2015), UNIDO (2013), UKCES (2014), United Nations (2010), von der Gracht and Darkow (2011), World Bank Group (2017)
Digital economy	Sharing economy, from an economy of goods to an economy of services	Boumphrey and Brehmer (2017), Grant Thornton (2017)
Financial Innovation	Digital currencies, cashless payment, the financial technologies (Fintech) revolution	Capgemini (2017), EY (2017)

### Social

Under the social dimension a key megatrend that identified is population growth. World population is forecasted to reach 8.5 billion by 2030 and the main contributors to this growth will be Africa and Asia followed by Northern America, Latin America and the Caribbean and Oceania (United Nations 2015). Consequently, demand for land and food will grow substantially by 2050. Another trend considers the ageing population as it projected that 56% of the total population will be 60 years or over by 2030, mainly in the developed regions (United Nations 2015). This will have an impact on the logistics industry as e.g. the average age of truck drivers has increased more rapidly in comparison with other industries due to the inability of attracting young workers entering the industry (The Conference Board of Canada, 2013). There is a shortage of 59,000 Heavy Goods Vehicle (HGV) drivers in the UK (FTA 2019). Apart from drivers, hiring skilled workers is a big challenge for supply chain managers as there is a shortage of workers with the right skills that delay also the implementation of digital technologies (MHI 2020). However, it is suggested that 35 million people will move from less developed countries to developed ones between 2015 and 2030 (Roland Berger 2017) that will lead to workforce expansion and therefore increasing demand and investment. New megacities and smart cities particularly will grow, and the related needed investments estimated to $1.8 trillion by 2030 (OECD 2015). By 2025 it is expected that more than 60% of the world population will be in urban cities (United Nations 2014).

These trends will lead companies to look for alternative transport modes that suit the urban centers and force them to change their overall distribution strategies with the ultimate goal to respond quickly to a rapidly changing and highly sophisticated consumer landscape (Öberg and Graham 2016). For example, the increasing demand for urban logistics led logistics providers to collaborate with retailers, which is called “re-logistification”. Another phenomenon is logistics sprawl i.e. relocation and concentration of logistics facilities towards suburban areas. Multi-floor logistics facilities (i.e. “flexible distribution centers whereby a fleet of mobile warehouses are parked at strategic locations throughout the city” will be utilized (CBRE 2017). Last but not least, there is a trend towards underground cargo transporters (DHL 2012).

In addition, consumption pattern will change due to various trends such as middle-class explosion and on-line shopping that will lead to the growth in the parcel market and in the last-mile delivery and reverse logistics services. These trends already led retailers to insource portions of their logistics function and build their own networks e.g. Amazon (PwC 2011). The recent pandemic also led consumers to panic buying and stockpiling; thus, supply chain managers faced the challenge with regards to demand and stockouts (Hall 2020). Due to the quarantine measures of the coronavirus crisis, several retailers such as Hennes & Mauritz AB, The Body Shop temporarily closed their physical stores which led online sales to increase. For example, online sales increased 49% between March 12-April 11 2020 in the USA; grocery (e.g. Instacart) and electric/electronics retailers are the most important online players while there only a few apparel companies that have been doing well e.g. Lululemon (Popper 2020). Thus, most of the customers are buing only essential items and according to Foolproof survey, 72% of adults in the UK supported that had either worn gloves or cleaned a public touch surface and 48% used contactless payment; thus, this pandemic will change the way that transactions are made and companies should adapt and use mobile payments, QR codes and completely touchless payments (Rigby 2020).

The new business models need to take into consideration the increasing social media engagement e.g. 68% of millennials tend to buy specific products or services based on friends’ social media (Nielsen 2016). Individualization has an impact on the production structures to be more decentralized e.g. healthy lifestyle led to demand for fresh, healthy, unprocessed foods with short shelf life (Mat et al. 2015). Technologies such as 3D printing will enable customers to get involved into the design phase as the customization of the products and services is an added value (Kudus et al. 2016). In the logistics sector, dedicated fleets are used to ensure service requirements are met. In Table 3 seven megatrends and 20 trends identified under the social dimension.

**Table 3:** Social dimension megatrends and trends

Megatrends	Trends	References
Population growth	Population boom in the developing countries, growing demand of resources (e.g. land)	Accenture (2016), Aggelakakis et al. (2015), Boumphrey and Brehmer (2017), Clausen et al. (2014), DHL (2013, 2016a), Harrington (2015), Kwiatkowski and Schäfer (2017), McKinsey Global Institute (2011), PwC (2012, 2016b), Roland Berger (2017), United Nations (2010), UN (2015), van Buren et al. (2016), Veolia (2016), WEF (2013)
Demographic change	Ageing population boom in developing countries, young population boom in developing countries, migration flows, labor shortages	Accenture (2016), ALICE (2013), Kearney ΑΤ (2016b), Boston Consulting Group (2016), Cranfield University (2017), Csiro (2016), BVL (2013), Deloitte (2017), DHL (2016b), European Agency for Safety and Health at Work (2016), Frost and Sullivan (2017), FTA (2016, 2017), Grant Thornton (2017), Kewill (2013), KPMG (2016), McKinsey Global Institute (2011, 2012), Miebach (2017), PwC (2012, 2015, 2016a), Roland Berger (2014), Sana (2017), SCM World (2014), SEMLEP Logistics (2013), Sikich (2017), SMMT (2015), Veolia (2016), von der Gracht and Darkow (2011), Wisskirchen et al. (2017), World Bank Group (2017)
Urbanization	Megacities, smart cities	Accenture (2016), Aggelakakis et al. (2015), Barclays (2014), Benjelloun et al. (2009), Bielefeldt et al. (2013), Boumphrey and Brehmer (2017), CBRE (2016, 2017), Citylab (2017), Clausen et al. (2014), Csiro (2014), Deloitte (2017), DHL (2012, 2013, 2016a, 2017), Elmegaard (2016), European Agency for Safety and Health at Work (2016), Gernandt (2012), Grant Thornton (2017), Harrington (2015), Invata (2017), ITC (2017), KPMG (2016), Mehmood et al. (2016), PwC (2009), Schuckmann et al. (2012), UN (2015), UNIDO (2013, 2016), United Nations (2010), Veolia (2016), von der Gracht and Darkow (2016), PLA (2016), PwC (2015, 2016b), Roland Berger (2017), World Bank Group (2017), WEF (2013, 2016)
Change in consumption pattern	Middle-class explosion, healthy diets and lifestyles, consumerism	Kearney ΑΤ (2014, 2016a), Barclays (2014), BVL (2013), Cerasis (2017), Csiro (2014), Deloitte (2015a, 2017), DHL (2013, 2017), Gernandt (2012), Horenberg (2016), Kewill (2013), Lee et al. (2016), PwC (2016c), Schmidt (2013), van Buren et al. (2016)
Individualism	New customer relationship, new shopping experience, increased customization	Kearney ΑΤ (2016b), Barclays (2014), Boumphrey and Brehmer (2017), BVL (2013), CBRE (2016), Cerasis (2017), Clausen et al. (2014), Csiro (2014, 2016), Deloitte (2015a, 2017), DHL (2012, 2013, 2016a), European Commission (2010), EY (2014, 2016), Gernandt (2012), Infor (2016), Invata (2017), KPMG (2016), Kwiatkowski and Schäfer (2017), McKinsey Global Institute (2012), PwC (2009, 2011, 2015, 2016a, 2017), Sana (2017), UNIDO (2016), Veolia (2016), WEF (2016), World Bank Group (2017)
Digital natives	Change of communication patterns, change of purchasing patterns, reshaping the workplace	PwC (2016b)
Knowledge based economy	Increasing demand for high-qualification jobs, emerging skills required, continuous learning culture	Business Europe (2011), Deloitte (2017), OECD (2015)

### Technological

Under the technological dimension digital transformation has emerged as a key megatrend. Technological advances have increased industrial productivity and there is a rise of new digital industrial technology known as Industry 4.0 which is powered by several technological advances such as big data analytics and robots (Hydrogen Council 2017). It is projected that big data and analytics software sales will grow to $70 billion by 2020 and they will be utilized in several industries (IDC 2017). For instance, companies in the pharmaceutical industry can utilize predictive analytics to detect demand spikes, stocks and delivery resources ahead of time (DHL 2017). Within the logistics industry, this trend can be applied for route optimization and real-time tracking of resources (CBRE 2017). Based on the survey from the Business Continuity Institute, most of the companies (59.6%) utilize big data analytics for their supply chain management to remove silos and enable them to have access to more comprehensive set of data (Business Continuity Institute 2019).

The digital transformation forces companies to rethink what customers value most and adjust operating models to achieve competitive advantages. For example, it is expected that 36% of financial services firms will make substantial investments in blockchain in the next three years (Harvey 2017). Blockchain can be used in supply chains to avoid paperwork processing in ocean freight, identification of counterfeit products, minimizing the inefficiencies in last-mile deliveries, and tracking of origin (Hackius Petersen 2017; PWC 2019). Regarding the megatrend technology development and automatization, the use of robots in factories and cyber physical systems will affect manufacturing companies in terms of production systems (Leitao et al. 2015). Developments within robotics enable ‘reshoring’ activities: reintroducing domestic manufacturing to a country. For example, Adidas sportswear manufacturer uses “intelligent robotic technology” and brought parts of their shoe production back to Germany from China.

Apart from robots, additive manufacturing enables easier prototyping and product development and move manufacturing activities closer to the final customers. This technology will reform logistics facilities and the relationships with the suppliers who provide the materials (Chen 2016). It is expected that 3D printing will reach a global value between $180 billion and $490 billion by 2025 (DHL 2016a). 3D printing will rise competition with e.g. small businesses that are funded through crowdsourcing (Mohr and Khan 2015). This technology will also impact the warehousing industry as along with the servitization some products can be stored digitally thus minimizing the inventory in the warehouses (Horenberg 2016). Regarding distribution, there will be a reduction in shipping, but it is important to highlight that raw materials are still needed (Chen 2016).

Distribution will be also impacted by drones that are expected to be used for quick delivery, thus minimizing the traffic on the roads, internal delivery and for tracking inventory (DHL 2014). Drones can play an important role in intralogistics, e.g. intraplant transport, but there are still issues such as regulations and public concern regarding the unmanned aerial vehicle (DHL 2014). Another important element is the autonomous vessels that can detect and adapt to changing sea and weather conditions and avoid collisions (KPMG 2015). There will be only three or four major liner companies, operating either as digitally enabled independents or as small units. Loading, stowage, and sailing will be fully autonomous to unloading directly onto autonomous trains and trucks and drones (McKinsey and Company 2017).

Transport electrification is another megatrend that is influenced by regulations regarding emissions and fuel efficiency. The main impacts on supply chain and logistics industries will be the use of battery electric vehicles, hydrogen fuel cell electric vehicles and hybrid vehicles. It is expected that 1 in 12 cars sold in Japan, California, South Korea and Germany would be powered by hydrogen by 2030 (Hydrogen Council 2017). Recently the UK government has announced £37 m of investment in developing electric cars, ships, and planes in the UK (UKRI 2020). There is not much availability and choice of electrified transport logistic vehicles in the logistics industry, but new materials lead to reduction in the weight of vehicles thus minimize fuel or electricity consumption (Kleiner et al. 2017). Last, renewable energy sources as a megatrend has several impacts on supply chain and logistics industries e.g. companies such as Apple aim to procure 100% of their electricity from renewable sources to minimize emissions from supply chain operations) (Apple 2018).

Thus, in Table 4 the four megatrends and 18 trends are presented.

**Table 4:** Technological dimension megatrends and trends

Megatrends	Trends	References
Digital transformation	Big data analytics, artificial intelligence, cloud-based computer systems, blockchain, Internet of Things	Gernandt (2012), BVL (2013), Clausen et al. (2014), Hajkowicz et al. (2016), DHL (2016b, 2017), Harrington (2015), PwC (2009), Schuckmann et al. (2012), von der Gracht and Darkow (2011), Cerasis (2017), DHL (2012, 2016a), FTA (2017), Grant Thornton (2017), PwC (2011, 2016a, 2017), Sana (2017), Kearney ΑΤ (2016a), Boumphrey and Brehmer (2017), Csiro (2014, 2016), Deloitte (2017), McKinsey Global Institute (2012, 2016a, 2016b), UKCES (2014), DHL (2015, 2017), Elmegaard (2016), European Agency for Safety and Health at Work (2016), WEF (2016), Kwiatkowski and Schäfer (2017), UN (2015), Veolia (2016), WEF (2013), Deloitte (2017), KPMG (2016), Miebach (2017), SCM World (2014), Sikich (2017), Wisskirchen et al. (2017), Barclays (2014), Bielefeldt et al. (2013), CBRE (2017), Mehmood et al. (2016), PLA (2016), UNIDO (2016), Lee (2016), von der Gracht and Darkow (2016), PwC (2016a), Schmidt (2013), Horenberg (2016), European Commission (2010), Infor (2016), PwC (2017), Boston Consulting Group (2015), Capgemini (2011), Deloitte (2013, 2016), EFT (2016), Heijster and Huijbers (2012), Jablonski (2016), PwC (2016a), Roland Berger (2017), Technopolis and Fraunhofer (2016), WEF (2017), Digital map (2017)
Technology development and automatization	Robots, cyber-physical system, augmented reality and virtual reality, 3D printing/additive manufacturing, drones, autonomous systems, automated guided vehicles, wearable devices	
Electrification of transport	Battery electric vehicles, hydrogen fuel cell electric vehicles, hybrid vehicles	FCH (2015), Hydrogen Council (2017), Markets and Markets (2015), Schorsch (2017), McKinsey (2010), Kleiner et al. (2017), Speranza (2018), Theverge (2016), S2R (2017)
Renewable energy sources	Production and storage of clean energy and application to transportation and industry, renewable energy for industrial processes	Hydrogen Council (2017), IGI Global (2017)

### Legal

Consumer protection laws are getting stronger so that consumers can easily return products free of charge or under warranty (Deloitte 2013) and companies have to recall products in case of problems towards customer’s safety. These trends impact the return process of supply chain. For instance, Toyota recalled 5.8 million vehicles because of faulty airbags. In addition, there are new EU privacy regulations that aim to protect any personal information. There are different directives regarding consumer protection in the digital market i.e. Directive 2011/83/EU on consumer rights, Directive 95/46/EC on the protection of individuals with regard to the processing and free movement of personal data.

Intellectual property law tries to secure the rights to inventions. However, for companies operating in certain countries such as China, it is challenging and there is a fear of security risks and loss of control when exchanging data (PwC 2016b). New emerging technology such as 3D printing can lead to several issues regarding the intellectual property, too; companies and governments need to solve any legal issue to enable technologies to contribute a large scale of open innovation (Chen 2016).

Thus, data exchange e.g. data regarding inventory, production processes, maintenance and the supply status of products or even financial data is a “big topic” and data sovereignty concepts need to be established for the protection of intellectual property. Data sovereignty is guaranteed by the following three aspects: (1) Data remain decentral and are not integrated in a joint data pool, (2) A precisely graded certification concept and (3) Security of infrastructure through new technological solutions (PwC 2016a). Last but not least, social and environmental regulations aim to achieve sustainable development through its activities on the environment as well as on employees and consumers (Tai and Chuang 2014).

Over the last decade, corporate social responsibility disclosure has increased dramatically. Moreover, vehicle electrification market is driven by stringent emission and fuel efficiency regulations. EU legislation set mandatory emission reduction targets for new cars sold in the EU specifically the fleet average should be 95 grams per kilometer by end of 2020 (Europa 2018). In addition to this, there is a target to reduce the greenhouse gas intensity of fuels by 6% by 2020 and maritime transport emissions levels at least 40% by 2030 (IMO 2018). The EU is setting the rules in order to boost resource efficiency and promote recycling e.g. EU’s Raw Materials Initiative. These regulations could lead to the relocation of carbon intensive companies and specific industries to regions with not so strict regulations (Ceniga and Sukalovaba 2015). The European Commission recently set a new circular economy action plan. More specifically there is a revision of EU waste legislation that includes new targets and processes to minimize packaging waste (e.g. harmonize separate collection systems), and “mandatory essential requirements” for packaging (i.e. reusable or recyclable) placed on the market (European Commission 2020). The review aims at restricting waste exports that could have harmful impacts in third countries.

Under the legal dimension three megatrends and nine trends emerged (see Table 5).

**Table 5:** Legal dimension megatrends and trends

Megatrends	Trends	References
Consumer protection laws	Cross-border payments, return products free of charge or under warranty, product safety regulations, privacy	Deloitte (2013), Durovic (2019)
Intellectual property law	Patents, data sovereignty	EvaluatePharma (2012)
Social and environmental regulations	Corporate social responsibility, emissions control regulations, waste and resources management regulations	Accenture (2016), Aggelakakis et al. (2015), Kearney ΑΤ (2016b), Citylab (2017), Deloitte (2013), DHL (2016a, 2012), ITC (2017), Heijster and Huijbers (2012), Kewill (2013), PwC (2011, 2015), van Buren et al. (2016), Veolia (2016), von der Gracht and Darkow (2011)

### Environmental

Pollution is a main trend when it comes to climate change. Air pollution, water pollution, and land pollution count to the major types of pollution. Focal firms are responsible for up to 80% of overall supply chain emissions so they are developing integrated approaches and strategies to minimize carbon emissions (Zimon et al. 2019). For example, Walmart, an American retailer, has launched a program to achieve a reduction of CO_2_ emissions from its Chinese suppliers by 50 million tons by 2030 (Reuters 2018). Furthermore, accidents, cancelation and delays due to atmospheric changes that are related with the increasing CO_2_ levels can lead to supply chain network disruptions. As pollution leads to increasing temperatures it will impact the transportation and logistics industries due to e.g. asphalt deterioration, buckling of rail lines and damage of the expansion joints on bridges and highways (Schwartz et al. 2014). Packaging will also be in the spotlight over the next five years as fast-tracked deliveries and frequent single-item shipments, create more carbon emissions and packaging waste (DHL 2019).

The depletion of resources such as water, oil (i.e. energy scarcity), and rare earth elements have been highlighted in many studies e.g. Bell et al. (2012). Resource scarcity, the second megatrend, is influenced by the global population, and the increasing demand for resources from consumers, regulations and geopolitical risks (Balatsky et al. 2015; Kalaitzi et al. 2018b). As population grows and the living conditions in developing countries rises along with changing eating habits, more people will require food or develop particular eating habits e.g. more meat, which in turn requires more water and agricultural area.

Specifically, global freshwater withdrawal by the manufacturing industry will grow more than 5% by 2050, over a year 2000 baseline, from 245 to 1552 billion m^3^ (Sachidananda et al. 2016). Companies, particularly the ones for whom water is the primary ingredient in their products, will face disruptions in their operations, e.g. Coca-Cola had to shut down factories in India due to social forces supporting Coca-Cola exploited water resources that were scarce in these regions.

Moreover, land scarcity is a big issue in densely populated cities, and logistics industry solutions have been trying to maximize land productivity e.g. innovative logistics hub construction. For example, a logistic hub will be completed in Singapore in 2019 integrating a multi-level inland container depot, a heavy vehicle park and warehouses (Mizar 2016). Apart from water and land, the energy demand worldwide is forecasted to increase; industrial energy demand will raise by 50% from 2015 to 2040 and the energy demand for the ships, planes and trains that carry products to factories and to markets will increase by 65% in the same timeline. Last but not least, rare earth elements scarcity is a crucial issue in manufacturing supply chains. Those elements are used in high technology-based products for different sectors, inter alia defense. China has 35% of global supply of the rare earth elements (Zhou et al. 2017) and applies tax and export quotas impacting the availability, supply and prices of those resources (Humphries 2003).

Another megatrend that emerged under the environmental dimension is catastrophic events/hazards e.g. flood, drought and pandemic. In particular there are three trends: natural disasters (like hurricanes, volcano eruptions), biological risks (like pandemic) and man-made disasters. Natural disasters can disrupt the supply chains causing interruption in trasportation and production processes: in Europe, recorded losses from climate extremes cost on average EUR 11.6 billion per year between 1980 and 2013 (European Environmental Agency 2017) and damages are projected to increase reaching 32 billion€ per year by 2050 (COACCH project 2018^1^). For example, after the earthbreak in Japan on 2011, the most important car manufacturers froze their production losing US$72 million a day (Arto et al. 2015); in particular, for several weeks, a shortage of over 150 parts left Toyota’s North American operations operating at 30% of capacity (Canis 2011). Regarding biological risks, it is possible to mention for example the spread of pandemic, supply chains should be mapped and restructured after the outbreak of SARS in 2003 but most of the companies thought that such a disruption is not likely to occur soon again and they were not willing to spend a large amount of labour and time required for these activities (Choi et al. 2020; Simpson 2020). Covid-19 outbreak in 2020 showed the importance of being proactive as this pandemic shut down temporarily factories (e.g. companies from the automotive and apparel industry), and led to traffic/ transportation restriction enforced by quarantine policy. Moreover, some countries enforced a ban to a few product categories e.g. Indonesia and Jordan banned imports of live animals from China and other countries saving vital medical equipment e.g. ventilators, face masks for their own citizens. It is forecasted that Covid-19 outbreak will cost the global economy at least $1trn (Simspon 2020). The last trend is related to disasters caused by human behavior; man-made disasters impact on supplier flow, warehousing, distribution and transportation. Ericsson in Spring 2001 lost $400 million just because of a fire in a supplier’s plant: the gaps of radio-frequency chips were supplied only by that supplier; it took pretty long for Ericsson to get aware of the situation and as a consequence the company had to give up its mobile phone business section (Munim et al. 2015). As consequence of an Explosion at BASF plant in Ludwigshafen in 2016, 15% of raw materials were missing for the entire supply chain and the production of some products at BASF has been stopped for many weeks (Ivanov 2018).

Under the environmental dimension three megatrends and six trends emerged (see Table 6).

**Table 6:** Environmental dimension megatrends and trends

Megatrends	Trends	References
Climate change	Pollution	Bielefeldt et al. (2013), Clausen et al. (2014), DHL (2013, 2012), FTA (2016), European Environmental Agency (2012), Accenture (2016), ITC (2017), PwC (2015), Mehmood et al. (2016), PLA (2016), Planning Institute Australia (2016), PwC (2009), Roland Berger (2016), Schwartz et al. (2014), United Nations (2015, 2010), UNIDO (2013), WEF (2013)
Resource scarcity	Lack of resources such as water, land, energy, food and rare earth elements, waste increase	Accenture (2016), Aggelakakis et al. (2015), ALICE (2013), Kearney ΑΤ (2014, 2016a), Barclays (2014), Boumphrey and Brehmer (2017), CBRE (2016), Cerasis (2017), Clausen et al. (2014), Csiro (2014), Csiro (2016), Deloitte (2013), DHL (2012, 2015, 2016a), European Commission (2010), Harrington (2015), Frost and Sullivan (2017), FTA (2016), Industry Innovation council (2011), Invata (2017), Kewill (2013), KPMG (2016), PLA (2016), Planning Institute Australia (2016), PwC (2009, 2011, 2015, 2016b), Roland Berger (2016), Schuckmann et al. (2012), United Nations (2010), van Buren et al. (2016), Veolia (2016), von der Gracht and Darkow (2011, 2016), WEF (2013, 2016)
Catastrophic events/hazards	Natural disaster, biological risks (e.g. pandemic),man-made disasters	Doherty and Botwright (2020), FTA (2020), Rogers and Oak (2020), Simpson (2020)

## Conclusion

Megatrends are reshaping society, economies, politics and the environment; so, they can be used to forecast future supply chain developments. There are only a few academic papers and reports that focus on specific megatrends in the field of supply chain management (i.e. Bowersox et al. 2000; Gernandt 2012; Jiang et al. 2017; Mat et al. 2015; Schuckmann et al. 2012; Seppälä 2016; Schmidt 2013; Stank et al. 2015; Sweeney 2013; von der Gracht and Darkow 2011, 2016) and do not provide a comprehensive view on trends and megatrends. This research addresses that gap through an extensive systematic review of the literature. The main contribution of this research is the comprehensive review and assessment of megatrends which was based not only on scientific publications, but also on sectoral studies, and governmental reports while, in addition, further insights have been collected and merged on the megatrends/trends within an interactive session. The need to expand the review and include a variety of publications (e.g. reports by consulting companies) was identified as a research opportunity in the literature review conducted by Malik and Janowska 2018. Based on a systematic literature review and a workshop, a total of 23 megatrends (M) and 72 related trends (T) were identified as the summary in Table 7 shows. To our knowledge this is the first published comprehensive literature review on megatrends and trends in the field of supply chain management.

**Table 7:** Overall megatrends and trends identified

Dimensions	Megatrends	Trends
Political	Protectionism	Import tariffs
		Quotas
		Different tax structures
		Subsidies
	Political stability	Terrorism/conflict
		Social unrest
	Supranationalism	Trade agreements
		Free movement
Economic	Global trade shift	Economic growth in emerging economies
		Export growth
		Investment
		Globalization
		Glocalization
		Emergence of born-global firms
	Digital economy	Sharing economy
		From an economy of goods to an economy of services
	Financial innovation	Digital currencies
		Cashless payment
		The financial technologies (Fintech) revolution
Social	Population growth	Population boom in the developing countries
		Growing demand of resources (e.g. land)
	Demographic change	Ageing population boom in developing countries
		Young population boom in developing countries
		Migration flows
		Labor shortages
	Urbanization	Megacities
		Smart cities
	Change in consumption pattern	Middle-class explosion
		Healthy diets and lifestyles
		Consumerism
	Individualism	New customer relationship
		New shopping experience
		Increase customization
	Digital natives	Change of communication patterns
		Change of purchasing patterns
		Reshaping the workplace
	Knowledge based economy	Increase demand for high-qualification jobs
		Emerging skills required
		Continuous learning culture
Technological	Digital transformation	Big data analytics
		Artificial intelligence
		Cloud based computer systems
		Blockchain
		Internet of Things
	Technology development and automatization	Robots
		Cyber-physical system
		Augmented reality and virtual reality
		3D printing/additive manufacturing
		Drones
		Autonomous systems
		Automated guided vehicles
		Wearable devices
	Electrification of transport	Battery electric vehicles
		Hydrogen fuel cell electric vehicles
		Hybrid vehicles
	Renewable energy sources	Production and storage of clean energy and application to transportation and industry
		Renewable energy for industrial processes
Legal	Consumer protection laws	Cross-border payments
		Return products free of charge or under warranty
		Product safety regulations
		Privacy
	Intellectual property law	Patents
		Data sovereignty
	Social and environmental regulations	Corporate social responsibility
		Emissions control regulations
		Waste and resources management regulations
Environmental	Climate change	Pollution
	Resource scarcity	Lack of resources such as water, land, energy, food and rare earth elements
		Waste increase
	Catastrophic events/hazards	Natural disaster
		Biological risks (e.g. pandemic
		Man-made disasters

The identified megatrends (e.g. globalization) could help managers and researchers alike to consider impacts on product, process and supply chain configuration. In particular, the study provides to managers in a systematic way megatrends and trends that are critical for the generation of future industrial scenarios and could offer a starting point to customise specific long-term strategies for their company. This chapter gives a glimpse into the opportunities and challenges that supply chain managers may face in the future; thus, they can develop an early warning system and develop as well as contingency plans. It provides an opportunity to prepare and respond to a broad range of potential disruptions in the future and to create more resilient and interactive supply chains. Based on these results, it is possible to formulate scenarios that describe the evolution and the implications of the identified megatrends and trends on supply chains for the next decade. Although the paper highlights some of the interesting insights in the SCL literature, there are a few limitations of the paper. Future research can consider other megatrends/trends given the dynamic, complex and fast evolving nature of modern supply chains.

## Footnotes

1 https://www.ecologic.eu/sites/files/publication/2018/2811-coacch-review-synthesis-updated-june-2018.pdf.
